# Genetics of Lifetime Reproductive Performance in Italian Heavy Draught Horse Mares

**DOI:** 10.3390/ani10061085

**Published:** 2020-06-23

**Authors:** Roberto Mantovani, Fabio Folla, Giuseppe Pigozzi, Shogo Tsuruta, Cristina Sartori

**Affiliations:** 1 Department of Agronomy Food Natural Resources Animals and Environment (DAFNAE), University of Padova, Agripolis, 35020 Legnaro (PD), Italy; max_fabio@libero.it (F.F.); cristina.sartori@unipd.it (C.S.); 2 Italian Heavy Draught Horse Breeders Association, 37068 Vigasio (VR), Italy; direzione@anacaitpr.it; 3 Department of Animal and Dairy Science, University of Georgia, Athens, GA 30602, USA; shogo@uga.edu

**Keywords:** fertility, lifetime, genetic parameters, mares, heavy breeds, horse, Italian Heavy Draught, fitness

## Abstract

**Simple Summary:**

Fertility is a very important fitness trait in animal production because of its significant economic importance, particularly in species with low reproductive efficiency. For this reason, it should be included as a breeding objective, particularly in horses, where the trait has received less interest than in other species. In this study, we attempted to identify a variable able to detect the lifetime reproductive performance of Italian Heavy Draught Horse (IHDH) mares and to analyze its possible implementation for breeding purposes, with the final aim to increase mares’ fertility efficiency. A phenotypic variable to be used early in life (at least after 3 breeding seasons) has been identified and validated in the study and its genetic component estimated. Results obtained indicated the proposed phenotypic measure of fertility as a good predictor of the lifetime reproductive success in IHDH mares and the variable showed high heritability (that is, the transmittable genetic component) for a fitness trait. Therefore, the use of the lifetime fertility rate proposed in this study for breeding purposes seems feasible, although some limitations could occur in the accuracy of individual breeding value estimates of mares and stallions.

**Abstract:**

Our aims were to find a phenotypic variable to express mares’ lifetime reproductive performance after 6 breeding seasons (BS) in Italian Heavy Draught Horse breed (IHDH), and to estimate its heritability. At first, 1487 mares in a training dataset were used to implement and validate a set of predictive coefficients (LFR-C) or equations (LFR-E) to estimate a lifetime foaling rate (LFR) after 6 BS, i.e., the number of foals generated divided by the opportunities to do so. Then, 3033 mares in a dataset with at least 3 registered BS, was used to estimate LFR for mares with 3, 4, or 5 registered RS. This dataset contained actual (*n* = 1950) and estimated (*n* = 1443) LFR, obtained by LFR-C, and LFR-E; Arcsine transformation of LFR-C and LFR-E were also analyzed in single trait animal models to estimate heritability. Overall, the LFR showed a moderate but significant genetic variation, and the heritability of the trait was high (0.24) considering it is a fitness trait. The arcsine transformation of LFR did not show any improvement of heritability. The present study indicates the possible use of a linear LFR variable for breeding purposes in IHDH breed considering both complete and incomplete reproductive careers.

## 1. Introduction

Fertility has a well-recognized role in animal production for its implication on the economic efficiency of the whole productive system, independently from the species considered [1,2,3]. In the last decades, many studies have been focused on cattle fertility as possible breeding goal both in the beef [4,5,6] or, more recently, in the dairy sector [7,8,9]. However, fewer studies on fertility have been carried out in horses as compared to cattle. Most researches in the horse industry has dealt mainly with the optimization of subfertility problems that occurs both in stallions [10] and mares [11]. Extensive reviews have been produced aiming at investigating the relationship between reproduction success and management [12], nutrition [13] (pp. 341–366), or genetics [14]. In addition, retrospective studies carried out at population level analyzing reproduction layouts [15,16] or on factors affecting horse births [2] are available in the literature for this species. However, little literature is available on the use of fertility traits in horse for breeding purposes as compared to cattle, particularly with beef cattle. As examples of the few studies in horses, genetic components for foaling rates were studied in Polish Warmblood horses [17], in Standardbred trotters and Finnhorses [18], also considering the effect of the inbreeding on fertility. The liability of being pregnant was studied in Thoroughbred horses [19] and the lifetime reproductive efficiency in Arabian broodmares [20]. Beef cattle shares with horses the common characteristics of a strong seasonality, in spite of a different reproductive efficiency, timing of ovulation, insemination protocols, and gestation length [12]. Following the review of Cammack et al. [21], focused on heritability of reproductive traits in beef cattle, there is no easy definition for fertility traits. This fact depends on a large number of factors affecting the reproduction success in both sexes. In addition, many fertility traits analyzed for breeding purposes have shown a generally low heritability [21], indicating a weak additive genetic component or, more often, a great residual variance. Earlier, Mayer et al. [4] showed a generally lower heritability in fertility traits repeated in subsequent breeding seasons like the calving success at insemination, rather than in those pertaining to the lifetime of an individual, like the number of calving. From a genetic point of view, Ponzoni et al. [22] confirmed the better results obtainable through the use of a lifetime fertility trait as the calving rate (i.e., the number of calves divided by the number of opportunities to calve, that is the efficiency of a female), in comparison to a trait related to a breeding season like the calving date (i.e., the day of the year in which a cow calves). However, some deficiencies can be attributable to the use of lifetime fertility traits, particularly the large amount of time necessary to estimate individual breeding values [4,22], delaying selection choices, and reducing genetic progress. For this reason, but also for the greater economic value of the calving date, this latter trait has been suggested as more suitable for breeding for fertility in beef cattle. Earlier calving dates are associated with greater weaning weight [21], but the economic advantage is not always perceptible in horse breeding. Indeed, foals sold at the end of the breeding season are not necessarily evaluated on the basis of weight, but on forelimb conformation and health status [23]. However, coldblood horses sold for meat production have more in common with the beef cattle production system, and the weight of weanlings could be considered attractive for animal breeding decisions because of its clearly identifiable economic value. On the other hand, the use of foaling date (i.e., the day of the year on which the mare foals) as possible fertility trait in mares has some possible drawbacks. For instance, some limitation for a correct comparison of mares’ reproductive performances could be due to the fact that the breeding seasons do not start at population level on the same date. Moreover, the joining period (i.e., start of the breeding season), is not well documented in many situations, lacking in pasture rearing systems. In addition, the gestation length in mares can easily lead to unwanted absence of conception (i.e., open mares) [16], increasing the possible number of missing information on subsequent breeding seasons. Therefore, lifetime fertility traits based on the reproductive success of mares could be probably more desirable and of easier use and comprehension for breeders. Mimicking the proposal of Meyer et al. [4], a lifetime foaling rate (LFR) could be defined as the number of foals produced by a mare divided by the number of opportunities to do so. Such trait could well represent a measure of the efficiency of reproduction of a mare, although with known limits, i.e., possible asymmetrical distribution due to the proportion variable, or the need for a sufficient number of available records per mare. Additionally, longer lifetime can increase opportunities for foaling but also the chance of failure, and older mare could express lower ratios values than younger animals. Moving from these points, this study has aimed at analyzing lifetime reproductive performance in Italian Heavy Draught Horse (IHDH) mares, and particularly at (i) identifying and validate a phenotypic variable useful to define a measure of IHDH mares’ lifetime fertility, and (ii) to analyze the genetic component, genetic trends and rank correlations between EBVs obtained in different ways. Specifically, 2 different method to estimate an LFR phenotypic value at a given lifetime endpoint in IHDH mares were considered, as well as 2 arcsine transformation of the same LFR variables, because of the known possible problems due to the use of ratio variables. The study was performed with a final aim to evaluate how to include LFR as a breeding goal for fertility in IHDH, suggesting a possible application also in other horse breeds.

## 2. Materials and Methods

### 2.1. Study Subject and Organization of the Study

The study was carried out on Italian Heavy Draught Horse (IHDH), a native breed originated in middle 19th century from crosses of French Breton stallions with local mares (North-East of Italy). In the middle of 20th century the meat attitude was added to the original heavy draught attitude, still important for some leisure activities and the agricultural work in organic farms. Nowadays the population includes 353 stallions and 2962 mares for a population of 5137 individuals (www.fao.org/dad-is/; update: August 2019). Genetic improvement is based on the evaluation of linear type traits scored in 6-months-old animals at a population level [24].

All data used in the study were obtained from the reproductive events officially registered at the studbook of the IHDH breed. Reproductive events report what happened to a mare at a target breeding season, including foaling or not. The breeding season is the moment of the year in which that reproductive event occurred. The study was carried out in two steps. In the first step, a training dataset was used in order to develop and compare alternative projection methods to estimate the foal production at the 6th breeding season in order to obtain a lifetime foaling rate (LFR) expressed as number of foals produced after 6 breeding season divided by the foaling opportunities, i.e., 6. The choice of the 6th breeding season was established as the endpoint representing a successful reproductive career for an IHDH mare, i.e., at about 9–10 years of age. Further breeding seasons were not considered. The breed, indeed, has a mean age at first foaling of 3.5 years and an average lifetime career of 9.3 years, with an average reproductive career of 5.8 years [16,25]. A different amount of breeding seasons could be considered in other breeds depending on their longevity. In a second step, a full data set was obtained in order to estimate the variance components for the LFR obtained by applying the two projection methods (then labeled “Coefficients” and “Equations”) developed at step 1 for an incomplete career with respect to the 6th breeding season. From this latter dataset, EBV were also obtained and both genetic trends and rank correlations were compared considering the 4 different expression of the LFR depending on the use of predictive coefficients or equations for incomplete career, and from the 2 arcsine transformed LFR (i.e., arcsine LFR obtained from coefficients or from equations projection methods). The characteristics of both datasets used in the study are reported in Table 1.

### 2.2. Training Dataset and Analysis

The training dataset (step 1) contained all reproductive events available from the studbook database for mares born after 1990. To enter the dataset, mares were required to hold an amount of 6 subsequent registered breeding seasons, to belong to environmental units by year of birth with at least 2 observations and to have both parents known. Environmental units considered were groups of farm-studs in the same geographical area (North-Italy; Centre-Italy; South-Italy) and common rearing system (Stable; Semi-wild; Wild). These divisions were done on the basis of specific characteristics recognized for horse management [16]. Environmental units were used instead of the stud-farm effect because of the low number of observation available considering the stud-farm effect. Indeed, the average number of mares/stud-farm is 3.3 in the IHDH population. The registered breeding season accounted for three different reproductive events: foaling, abortion, or involuntary absence of conception (i.e., open mares) in subsequent calendar years. The data editing provided also the discard of mares from dataset if they had a first registered events when aged <3 years or >4 years or when the interval between foaling resulted <11 or >17 months, as in previous studied on the same population [16]. At the end of the editing process, 1487 mares were retained for the subsequent analysis that consisted in obtaining a set of predictive coefficients or equations (by means of the GLM or REG procedure of SAS, respectively; SAS^®^ statistical software; SAS^®^ version 9.4, SAS Institute Inc., Cary, NC, USA) allowing the estimates of the no. of foals produced at the 6th breeding season depending on the basis of (i) the previous no. of foals after either 3, 4, or 5 breeding seasons, and (ii) the age at first foaling (3 or 4 years; Table 1). A lifetime foaling rate (LFR) was then obtained for each mare by dividing the no. of actual and predicted foals at the 6th reproductive events for the no. of opportunities of doing so (i.e., 6). The predictive ability of coefficients or equations was analyzed by comparing the actuals and the predicted values expressed for each mare using the following statistics:

1. the percentage squared bias (PSB; [26]), obtained from the formula:
  PSB = 100 (**y** − **ŷ**)’ (**y** − **ŷ**)/(**y**’ **y**) (1)
  where **y** is a vector of actual and **ŷ** is a vector of predicted values;
2. the mean absolute deviation of residuals (MAD; [27]) calculated from the formula:
  MAD = Σ |(**y** − **ŷ**)|/*n* (2)
  where |**y** − **ŷ**| are absolute differences between actual (**y**) and predicted values (**ŷ**), respectively, and *n* is the number of observations;
3. the standard deviation of residuals obtained as **y** − **ŷ**, where **y** and **ŷ** are vectors of actual and predicted values, respectively.

### 2.3. Full Dataset and Analysis

A full dataset (step 2) was obtained considering all mares that had at least 3 registered breeding seasons, born after 1990, located in environmental units with at least 2 observations, and with both known parents. The threshold of 3 breeding seasons was chosen to have robust data to predict the LFR at the 6th event (1 or 2 events are too few to obtain robust prediction). The editing process followed the same rules as for the training dataset, and at the end of editing, LFR was calculated for 3033 mares applying either coefficients or equations methods for mares with 3, 4 or 5 registered breeding seasons. The final dataset consisted of a mixture of actual (*n* = 1950) and predicted (*n* = 1443; Table 1) LFR both treated as linear variable or arcsine transformed as in Meyer et al. [4] on the basis of suggestion given for proportion variable by Fernandez [28]. Therefore, 4 different LFR variables were considered: 2 of them obtained using the predictive methods of Coefficients (LFR-C) or Equations (LFR-E) as explained above, and 2 arcsine transformations of LFR-C and LFR-E). For each variable normality of distribution and preliminary ANOVA (UNIVARIATE and GLM procedure, respectively; SAS^®^ statistical software; SAS^®^ version 9.4, SAS Institute Inc., Cary, NC, USA) were carried out. The latter one was run to establish which non-genetic effects could be taken into account in the genetic model. Some preliminary genetic models with increasing complexity were also run to see the variations in heritability when different animal-based factors were included (Table S1). The final and best-fitted model (AIC criterion [29]) was the one with the greatest complexity. Among the non-genetic fixed effects that accounted for a significant part of the total variance there were the above-mentioned environmental unit by birth year (EU-BY, 125 levels, including 2 to 82 records; Table 1), and the age first foaling (AF; 2 levels, 3 or 4 years; Table 1). The final model also included the effect of individual inbreeding as covariate, calculated on the whole studbook data updated at December 2019 using a recursive algorithm able to recover the incomplete lineages in pedigree [30]. Moreover, both additive and non-additive genetic effects were considered, the latter in terms of dominance effect. The matrix notation for the final single trait animal model genetic analysis can be written as follows:

**y** = **Xβ** + **Z_h_h** + **Z_a_a** + **Z_d_d** + **e** (3)

where **y** is an N x 1 vector of observations, **β** is the vector of systematic fixed effects of order **p** (AF and the linear covariate for inbreeding), **h** is the vector for the random effect of order **q** for EU-BY, **a** is the vector of random animal effect of order **q** (6801 animals in pedigree file, i.e., tracing back up to 12th generation for mares with records; Table 1), **d** is the vector of random dominance effect (9413 levels) and **e** is the vector of residual effects. Furthermore, **X**, **Z_h_, Z_a_** and **Z_d_** are the corresponding incidence matrices with the appropriate dimension. The assumptions about the structure of (co)variance were as follows:

(4) $$\mathrm{Var}(h)=I\sigma_{h}^{2},\;\mathrm{Var}(a)=A\sigma_{a}^{2},\;\mathrm{Var}(d)=D\sigma_{d}^{2},\;\mathrm{Var}(e)=I\sigma_{e}^{2}$$

where $\sigma_{h}^{2}$ is the herd variance, $\sigma_{a}^{2}$ is the additive genetic variance, $\sigma_{d}^{2}$ is the non-additive dominance variance, $\sigma_{e}^{2}$ is the residual variance, **A** is the numerator additive relationship matrix, **D** is the dominance relationship matrix, and **I** is an identity matrix.

Variance components were estimated using the AIREML software from the BLUPF90 family [31]. The dominance relationship matrix was previously built using the RENDOMN software of the same developers [31]. Phenotypic variance was calculated as the sum of all the variances included in the model. Heritability values were obtained for normal or arcsine transformed LFR (Coefficient and Equations methods) with the classical formula of Falconer [32] (p. 163), and the standard errors of the heritability (${\mathrm{SE}\;}_{h^{2}}$) were computed applying the formula of Lynch and Walsh [33]:

(5) $${\mathrm{SE}\;}_{h^{2}}{=h}^{2}\times {\left(\frac{{\mathrm{Var}(\sigma }_{a}^{2})}{{{(\sigma }_{a}^{2})}^{2}}+\frac{{\mathrm{Var}(\sigma }_{p}^{2})}{{{(\sigma }_{p}^{2})}^{2}}+\frac{{2\mathrm{Cov}(\sigma }_{a}^{2}{,\sigma }_{p}^{2})}{\sigma_{a}^{2}\sigma_{p}^{2}}\right)}^{2}$$

where h^2^ is heritability of the trait, $\sigma_{a}^{2}$ and $\sigma_{p}^{2}$ are the additive genetic and phenotypic variances of the trait, Var($\sigma_{a}^{2}$), Var($\sigma_{p}^{2}$) are their respective predicted error variances, and Cov($\sigma_{a}^{2}{,\sigma }_{p}^{2}$) is the predicted error (co)variance.

Breeding values obtained for all different LFR expression were estimated for all animals in the pedigree. Standardized EBVs considering the mean EBV of recorded mares born in year 2000 and the genetic standard deviation of the trait were obtained. The year 2000 was chosen to set the genetic basis (the year with mean EBV equal to zero) in an intermediate birth year. Rank correlations analysis for mares with record (*n* = 3033) and for stallions (*n* = 77) that showed a minimum accuracy of 0.65 (i.e., a minimum of 9 recorded daughters) calculated with the formula reported in Mrode [34] (p. 44–46) for EBV from progeny records, were carried out. Annual genetic trends were also generated from standardized EBVs using as last reference birth years the year accounting for >100 contemporary mares with records or >15 contemporary stallion sires of mares with records. From this last reference birth year (i.e., 2012 for mares; 2008 for sires), trends were generated by tracing back the average breeding value belonging to animals born in 15 adjacent years.

## 3. Results

### 3.1. Validation of the Phenotypic Variable to Measure Lifetime Fertility

Validation coefficients calculated to measure the predictive ability of two projection methods for incomplete career of IHDH mares, grouped by the number of known breeding seasons, are presented in Table 2.

As expected, all coefficients showed a progressive reduction, i.e., better fitting, when the timeline for projection was reduced, i.e., from the 3rd to the 5th registered breeding season. Looking at the average predictive ability, PSB, MAD, and SDR statistics resulted lower when the projection was carried out by means of the linear regression (Equations method) as respect to the use of GLM coefficients (Table 2), i.e., the LFR-E allowed minimum bias [26] and reduced the residuals between actual and predicted LFR [27].

Table 3 shows the descriptive statistics obtained on the full dataset, i.e., after the application of the predictive coefficients or equations on foals production of all mares with less than 6 registered breeding seasons, and after the extrapolation of the LFR in different scenarios accounting also for arcsine transformation of LFR-C or LFR-E.

**Figure 1:**
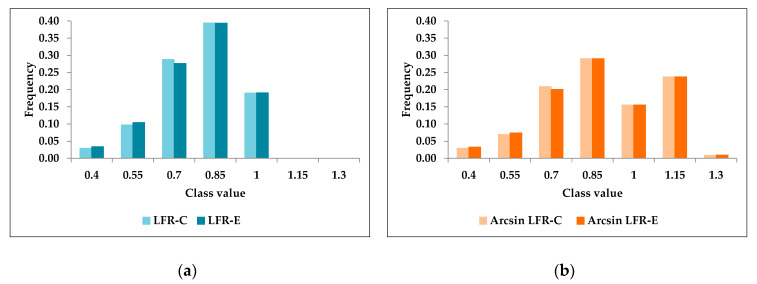
Class distribution of lifetime fertility rate (LFR) obtained in the full dataset (*n* = 3033) combining actual and predicted foals after the 6th breeding season; In (**a**) distribution of linear LFR obtained by predicting incomplete reproductive career with coefficients (LFR-C) or equations (LFR-E); in (**b**) normal distribution of arcsine transformed LFR obtained by predicting LFR with coefficients (Arcsine LFR-C) or equations (Arcsine LFR-E).

In both cases, the arcsine transformation of LFR produced an increase in both the mean and standard deviation as compared to the linear LFR-C or LFR-E (Table 3). The use of LFR-C produced, both as linear and arcsine transformed variable, a negative index of skewness, i.e., greater density on the left side of the distribution (−0881 and −0.506 for LFR-C and Arcsine LFR-C, respectively). On the other hand, LFR-E and its arcsine transformation determined a longer or fatter right side of the distribution (Table 3; Figure 1). However, in both cases, the arcsine transformation of the two LFR values produced a shift toward the right side of the distribution and lower peaks (i.e., lower kurtosis, Table 3 and Figure 1).

However, all variables resulted normally distributed as confirmed by significant coefficients of the statistics produced (Table 3). Therefore, all subsequent analyses applied have been carried out complying the specific distributional assumptions at the basis the mixed model, although it has been reported that many statistical techniques based on normality assumption are more robust than the assumption itself [35].

### 3.2. Genetic Analysis of Lifetime Fertility

The genetic analysis carried out on the 4 different expression of the LFR is reported in Table 4.

All LFR have shown a detectable genetic variation, although greater for the arcsine transformed LFR as compare to the linear LFR (8.9 on average vs. 4.8, respectively). However, also the herd, dominance and residual variance resulted greater for the arcsine transformed variables, leading to almost similar heritability estimates, i.e., about 0.24. The ratio of the dominance variance on the total phenotypic variance was similar in all the models and was on average 0.074. The inclusion of this non-additive genetic components allowed to slightly decrease the heritability of 0.02, as noticed in preliminary analysis (Table S1). However, the AIC [29] resulted lower when the LFR were obtained through the equation (LFR-E) based estimates of the foal production at the 6th breeding season for mares with incomplete reproductive career. Again, as analyzed in the training dataset, this method produced more suitable results.

The use of a linear value for LFR or its arcsine transformation did not affect the rank correlation both in groups of mares and in stallions accounting a homogeneous accuracy (Table 5) and did not modify the estimates of the genetic trends (Figures S1 and S2). Genetic trends completely overlapped in mares and with small intersections in stallions’ sires of the mares with phenotypic LFR.

## 4. Discussion

A low fertility with respect to other livestock species is well known in horses and may have a negative impact on the profitability of breeding [18,19]. A major weakness in reproduction is the strong impact of management decisions such as the time or the method on insemination on the measured traits [2,18]. Management can affect fertility more in sport breeds than in draught or meat breeds, e.g., due to the wider use in sport horses of frozen semen for mating, that may reduce indeed the chances of successful conception [2]. Conversely, in draught and meat horses natural mount is common: in Italian Heavy Draught Horse, e.g., it constitutes about 90% of mating. Moreover, in sport horses there can be the choice to delay the age at first parity to favor the participation in sport competitions, despite older mares being less fertile [2]. Again, horse breeders are often amateurs, and they do not choose the Spring as the best season to conceive due to physiological factors [2]. Fertility of horses can be also affected by animal-based factors, such as physiological traits, inbreeding, and genetic factors [18]. Sperm quality, e.g., can be very variable due to stallion-specific genetic and non-genetic effects [36]. It is well known that inbreeding depression mainly occur on fitness traits like fertility and longevity [2,18,19], and for this reason, genetic analyses on fertility traits generally include the inbreeding effect [18]. Finally, both additive and non-additive effects may affect fertility, depending on the population structure, and considering a dominance relationship matrix within the genetic analysis may prevent an inflated estimation of the heritability of the trait [27].

The lifetime fertility rate of the present study has been developed in IHDH, but it can be easily transferred to other horse breeds by looking at their average reproductive career and using this value as a denominator of the ratio. The choice of this LFR as a trait is due to the fact that it allows one to consider the non-foaling events occurring in a mare career to find out the mare reproductive efficiency, as well as to predict the efficiency after just some years of a career (at least 3) thanks to the use of prediction equations. The phenomenon of a better fitting due to the LFR-E, i.e., linear regression, as compared to coefficients (LFR-C) is probably due to the different properties of linear equations as respects to the classification factors. Notwithstanding, the difference observed in this study between the coefficients and equation predictive methods resulted in general very small. Comparable criteria based on residuals analysis have been previously carried out in comparison to predictive methods or models. For example, Albertsdóttir et al. [37] used the R method [38] to compare the estimation bias of breeding values for conformation and riding ability in Icelandic horses. Cross-validation methods for analyzing the predictive ability of different models in trotters [39] or, more recently in Spanish Purebred—Pura Raza Español [40] have been also reported. However, both R method and cross-validation have been applied in situations accounting for many thousands of records. Additionally, these methods have been focused on evaluating possible biases in EBVs or predictability of models after splitting randomly the dataset in two parts accounting different percentage of the whole dataset (i.e., training and validation datasets accounting for 75% and 25% of records, respectively). However, in the present study, neither the possible biases on EBVs were an objective of the study, nor the cross-validation could have been easily implemented considering the number of records available to predict the foal production at the specific endpoint. Therefore, classical coefficients proposed for phenotypes were applied as the best choice available.

The comparison of genetic parameters obtained in this study with other literature is not easy because there are few specific studies on horses. The study of Sairanen et al. [18] has dealt with equine fertility, but the trait analyzed was a foaling rate treated as a dichotomous variable considering the reproductive success of Standardbred and Finnhorse mares repeated over subsequent breeding seasons. Heritability estimates from this study resulted very low [18], ranging from 0.011 to 0.030%, suggesting that breeding for fertility could not be considered a primary selection goal in the Finnish breeds analyzed. The study of Mucha et al. [19] on Thoroughbred horses also used a dichotomous variable about the reproductive success or not, and applied different approaches, obtaining heritability values ranging from 0.06 to 0.14. An approach similar to the present study was applied by Sabeva and Apostolov [20] on Arabian broodmares. Here, a born foals index was defined as 1 plus the ratio between the number of foals on the longevity, that provided a heritability of 0.27. This value is similar to the heritability estimates reported in the present study. Additionally, another an available comparison of the studied LFR is with the calving rate refereed to lifetime analyzed by Meyer et al. [4] in beef cattle. Nevertheless, the study of Meyer et al. [4], reported lower heritability than the values estimated in the present study, ranging from 0.02 to 0.17 depending on the breed considered. However, the variable used [4] was not defined at a specific endpoint as in the present study, and the number of opportunities (with maximum value of 11), was accounted in the model as a fixed class factor. On the other hand, the main differences with the estimates of Meyer et al. [4] are in the proportional amount of the residual variance with respect to the additive genetic component. Such changes could be partly due to a general reduction of variability caused by the definition of a specific endpoint for the reproductive career. The raw standard deviation of LFR in the full dataset was indeed about half the value reported on average for cattle and zebu crosses by Meyer et al. [4], i.e., 0.143 vs. 0.314. Also, the phenotypic variance estimated in the present study resulted lower than that reported by Meyer et al. [4], i.e., 19.69 vs. 69.50, respectively. In spite of such occurrences, the heritability estimates of the present study were much greater than those reported in cattle [4], allowing the deduction that genetic determinants of reproductive potential are greater in horses than in cattle or zebu crosses. As in Meyer at al. [4], the arcsine transformation of the ratios did not produce changes in the heritability estimates, which remained very close to the linear LFR values. In general, moderate-low heritability has been reported for fertility traits in cattle by many authors [6,17,41]. However, for traits as age at first calving [42], heifer’s pregnancy [43,44], heifer’s puberty [45], and number of calves [46], heritability values between 0.20 and 0.30 have also been reported for beef cattle. In general, as pointed out by Cammack et al. [17], a wide range of heritability values have been reported for the same fertility trait in beef cattle, independently referred to different breeding season or expressed as lifetime fertility traits. This could be obviously due to the different models implemented, the quality of data and pedigree recording, the number of available phenotypes, and the connectedness of data [47]. All of these factors have a recognized effect on the estimates of genetic parameters. Additionally, recent studies carried on horse longevity, another functional trait linked animal fitness, have shown heritability values ranging from 0.09 to 0.31 in Danish show jumping horses [48], and 0.20 in Pura Raza Español used for dressage [49]. Longevity and lifetime fertility are generally highly correlated in livestock, since brood mares are used to give births until the end of their career. These values indicate that horse have a different genetic potential as compared to cattle, particularly if used for sport activities like in the latter cases. Indeed, longevity in horses may depend greatly on the ability shown in sport [48,49].

In the present study, small amounts of data have been used in spite of the fact that all the available information at population level were accounted for. However, the estimated standard errors resulted generally low, allowing the deduction that our estimates were not affected by a reduced dataset size. Additionally, the consistency of the genetic parameters and their standard errors both as linear and arcsine transformation seem to indicate the absence of possible artefacts due to the distributional properties of the analyzed variables [4].

In general, the observed genetic trend was positive but almost steady in both mares and stallions, due to the absence of any selection process for fertility in the breed [17,25]. The almost absence of differences in trend among traits suggest that there are equivalent for selection purposes. The slight difference detectable after year 2005 could be related to low sample sizes. However, in spite of general good reproductive performances of the IHDH mares [17], a small but positive genetic trend for LFR seems promising for a further improvement of the stud-farms’ income. Additionally, the use of the LFR could become a tool for management practices addressed to an increase of the fertility rate for breeders, due also to the high heritability for a fitness trait. The only actual limiting factor that requires cautiousness is the use of EBVs obtained from predicted phenotypes. Indeed, prediction implies reliability lower than 1.0 and, as shown in this study, increasingly uncertainness was observed by increasing the timeline of prediction. Therefore, low reliability in the prediction leads to an overestimation in the accuracy of EBVs, with possible negative implications on the selection process. A possible penalization of the accuracy for predicted phenotypes could be then taken into account, together with a further analysis aimed at investigating genetic correlations obtained by analyzing the actuals and the predicted phenotypes.

## 5. Conclusions

In conclusion, the LFR variable calculated at a specific endpoint using actual and estimated number of foals seems a feasible method to express lifetime reproductive success in IHDH mares. The use of estimates of foal production at 6th reproductive event obtained through equations (regression analysis) performed slightly better than the use of coefficients (GLM analysis). No improvement in the estimates of genetic parameters and EBVs can be obtained by the arcsine transformation of the LFR. A significant genetic variation was detected for LFR, estimating a high heritability value for a fitness trait. In addition, a small but positive genetic trend was observed, although the breed has not been selected yet for LFR. In spite of a greater than zero heritability value, a careful use of the EBVs is suggested because the prediction leads to an overestimated accuracy for an individual with an incomplete reproductive career. Last, further analysis of the LFR is required for a final general validation of the projection method. Results of the study will allow for breeders to choose individuals with a greater reproductive efficiency for breeding purposes. This aspect is important in all horse breeds due to the low fertility of the species. Once the average length of the reproductive career has been defined, the LFR can also be applied in other breeds as a lifetime fertility trait available for selection.

## Figures and Tables

**Table 1:** Characteristics of the training and the full dataset used in the study.

Item	Training Dataset	Full Dataset
Mares with actual records, number (no.)	1487	3033
Mares with projected records, no.	-	1443
Environmental units ^1^ by birth year (EU-BY), no.	97	125
Mean records in EU-BY, no.	15.3	24.2
Age at first known breeding season, months (mo.)	43.7 ± 6.6	43.9 ± 6.5
3 years first foaling mares, mo.	36.6 ± 1.8	36.6 ± 1.8
4 years first foaling mares, mo.	49.1 ± 2.5	49.0 ± 2.4
Animals in the pedigree file, no.	-	6803
Sires of mares with record, no.	400	602
Dams of mares with record, no.	1011	1848
Daughters/sire	3.5	4.8
Daughters/dam	1.3	1.5

^1^ Environmental units are intended to be a group of farm-studs in the same geographical area and common rearing system of mares and foals.

**Table 2:** Predictive ability of coefficients or equations projection methods used to estimate the foals production at the 6th breeding season and to obtain the lifetime fertility rate (foal produced divided by the number of opportunities) starting from foals produced after 3, 4, or 5 known breeding seasons or considering the whole predictive ability of projections.

Item	Projection Method	
	Coefficients (LFR-C)	Equations (LFR-E)
Projection from 3 known breeding seasons		
- PSB ^1^	0.0604	0.0148
- MAD ^2^	0.1813	0.0793
- SDR ^3^	0.1028	0.0986
Projection from 4 known breeding seasons		
- PSB	0.0437	0.0092
- MAD	0.1561	0.0606
- SDR	0.0832	0.0784
Projection from 5 known breeding seasons		
- PSB	0.0319	0.0041
- MAD	0.1377	0.0350
- SDR	0.0622	0.0537
Average		
- PSB	0.0453	0.0094
- MAD	0.1584	0.0583
- SDR	0.0827	0.0769

^1^ PSB = percentage squared bias [26]; ^2^ MAD = Mean absolute deviation of residuals, [27]; ^3^ SDR = standard deviation of residuals. Descriptions of statistics are provided in the text.

**Table 3:** Descriptive statistics for lifetime fertility rate (LFR), normality tests (Kolmogorov-Smirnov D and Anderson Darling A-Sq parameters and significance), skewness, and kurtosis of the 3033 data in the full data set obtained combining actual and predicted number of foals after 6 breeding seasons and considering different prediction methods.

Statistic	LFR-C ^1^	LRF-E ^2^	Arcsine LFR-C ^3^	Arcsine LFR-E ^4^
Mean ± standard deviation	0.700 ± 0.142	0.699 ± 0.144	0.794 ± 0.195	0.793 ± 0.197
Kolmogorov-Smirnov D	0.16 (*p* < 0.01)	0.14 (*p* < 0.01)	0.15 (*p* < 0.01)	0.11 (*p* < 0.01)
Anderson-Darling A-Sq	82.9 (*p* < 0.01)	78.7 (*p* < 0.01)	67.2 (*p* < 0.01)	60.1 (*p* < 0.01)
Skewness	−0.881	0.144	−0.506	0.197
Kurtosis	0.485	0.986	−0.300	−0.004

^1^ LFR-C = predictive method for LFR based on regression coefficients; ^2^ LFR-E = predictive method of LFR based on equations; ^3^ Arcsine LFR-C = arcsine transformation of the LFR-C; ^4^ Arcsine LFR-E = arcsine transformation of the LFR-E.

**Table 4:** Results of genetic analysis carried out on the lifetime fertility rate (LFR) obtained combining actual and predicted number of foals after 6 breeding seasons and considering different prediction methods for incomplete reproductive career (by coefficients; LFR-C; by equations; LFR-E) and arcsine transformation of both the LFR-C (Arcsine LFR-C) and the LFR-E (Arcsine LFR-E).

Item	LFR-C	LRF-E	Arcsine LFR-C	Arcsine LFR-E
Herd Variance ^1^	0.141	0.160	0.259	0.276
Genetic Variance ^1^	4.848	4.693	8.975	8.829
Dominance Variance ^1^	1.628	1.358	2.896	2.573
Residual Variance ^1^	13.426	14.259	25.660	25.463
Phenotypic Variance ^1^	20.043	19.469	37.790	37.141
Heritability	0.242	0.241	0.238	0.237
SE Heritability	0.043	0.042	0.042	0.042
AIC	−3290	−3380	−1370	−1424

^1^ Multiplied by 10^3^.

**Table 5:** Rank correlation coefficients between standardized EBVs obtained for different expression of lifetime fertility rate (LFR ^1^) in Italian Heavy Draught Horse mares with records (*n* = 3033) or stallions with at a minimum accuracy of 0.65 (*n* = 77).

Comparison	Mares with Actual or Predicted LFR	Stallions with ≥9 Daughters with Actual or Predicted LFR
LFR-C ^2^ vs. LFR-E ^3^	0.997	0.993
LFR-C vs. Arcsine LFR-C ^4^	0.996	0.996
LFR-E vs. Arcsine LFR-E ^5^	0.996	0.995
Arcsine LFR-C vs. Arcsine LFR-E	0.997	0.993

^1^ LFR was obtained combining actual and predicted number of foals after 6th breeding season and considering different prediction methods for incomplete reproductive career; ^2^ LFR-C = prediction method for LFR based on regression coefficients; ^3^ LFR-E = prediction method of LFR based on equations; ^4^ Arcsine LFR-C = arcsine transformation of the LFR-C; ^5^ Arcsine LFR-E = arcsine transformation of the LFR-E.

## Supplementary Materials

The following are available online at https://www.mdpi.com/2076-2615/10/6/1085/s1, Figure S1: Genetic trends obtained from mean standardized EBVs by birth year in Italian Heavy Draught Horse mares with record (*n* = 2515) tracing back 15 years from the last birth year with a minimum number of 100 mares; Figure S2: Genetic trends obtained from mean standardized EBVs by birth year in Italian Heavy Draught Horse stallion sires of mares with record (*n* = 399) tracing back 15 years from the last birth year with a minimum number of 15 stallions; Table S1: Comparison of different models run on the lifetime fertility rate (LFR) obtained combining actual and predicted number of foals after 6 breeding seasons and considering different prediction methods for incomplete reproductive career (by coefficients; LFR-C; by equations; LFR-E) and arcsine transformation of both the LFR-C (Arcsine LFR-C) and the LFR-E (Arcsine LFR-E).
