# Supplementary material for: Genetics of Lifetime Reproductive Performance in Italian Heavy Draught Horse Mares
Source: Animals (Basel). 2020 Jun 23;10(6):1085. doi: 10.3390/ani10061085 (PMC7341281; doi:10.3390/ani10061085)
Supplement: Supplementary file 1 [file animals-10-01085-s001.pdf]

animals

MDPI

Supplementary Files: Genetics of Lifetime Reproductive Performance in Italian Heavy Draught Horse Mares

Roberto Mantovani, Fabio Folla, Giuseppe Pigozzi, Shogo Tsuruta and Cristina Sartori

Table 1. Comparison of different models run on the lifetime fertility rate (LFR) obtained combining actual and predicted number of foals after 6 breeding seasons and considering different prediction methods for incomplete reproductive career (by coefficients; LFR-C; by equations; LFR-E) and arcsine transformation of both the LFR-C (Arcsine LFR-C) and the LFR-E (Arcsine LFR-E).

Item	LFR-C	LRF-E	Arcsine LFR-C	Arcsine LFR-E
Model 1 (AF + EU-BY$_{fixed}$ + a + e) [1]				
Genetic Variance[2]	5.018	4.855	9.378	9.226
Residual Variance[2]	14.99	14.52	28.33	27.77
Heritability	0.251	0.251	0.249	0.249
AIC	−2847	−2939	−1006	−1062
Model 2 (AF + EU-BY$_{rnd}$ + a + e) [1]				
Herd Variance[2]	0.177	0.192	0.332	0.341
Genetic Variance[2]	5.289	5.069	9.652	9.459
Residual Variance[2]	14.82	14.41	28.22	27.71
Heritability	0.261	0.258	0.253	0.252
AIC	−3287	−3379	−1367	−1422
Model 3 (AF + EU-BY$_{rnd}$ + F + a + e) [1]				
Herd Variance[2]	0.168	0.184	0.313	0.324
Genetic Variance[2]	5.394	5.161	9.863	9.633
Residual Variance[2]	14.72	14.33	28.03	27.56
Heritability	0.266	0.262	0.258	0.257
AIC	−3288	−3379	−1368	−1423
Model 4 (AF + EU-BY$_{rnd}$ + F + a + d + e)[1]				
Herd Variance[2]	0.141	0.160	0.259	0.276
Genetic Variance[2]	4.848	4.693	8.975	8.829
Dominance Variance[2]	1.628	1.358	2.896	2.573
Residual Variance[2]	13.426	14.259	25.660	25.463
Heritability	0.242	0.241	0.238	0.237
AIC	−3290	−3380	−1370	−1424

[1] AF: age at first foaling; EU-BY: environmental unit by birth year, considered as fixed (BYfixed) or random (BYrnd); F: individual inbreeding; a: additive variance; d: dominance variance; e: residual error. [2] Multiplied by 10^3.

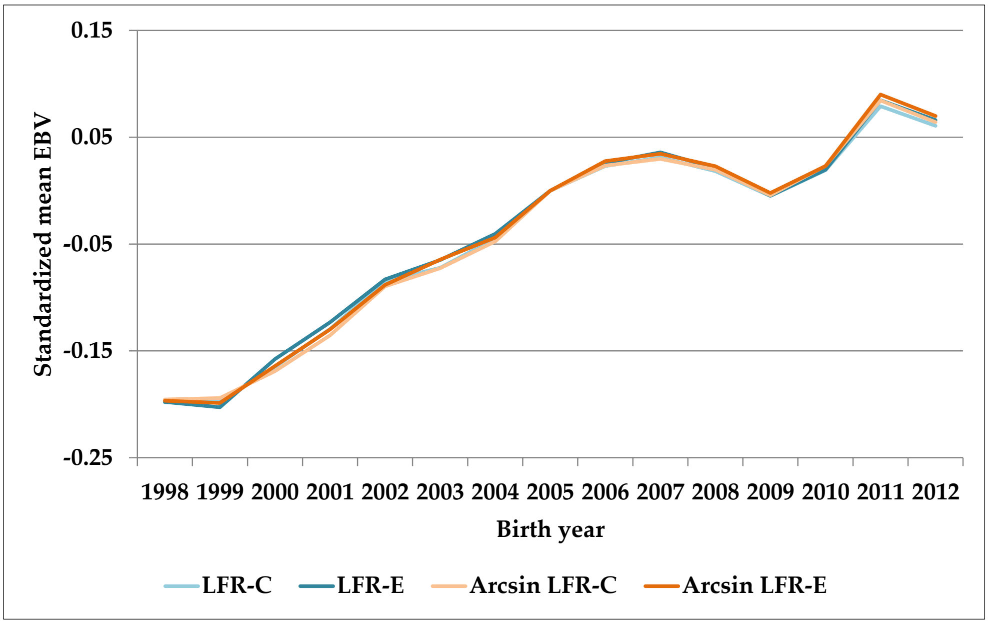

Figure 1. Genetic trends obtained from mean standardized EBVs by birth year in Italian Heavy Draught Horse mares with record (n = 2515) tracing back 15 years from the last birth year with a minimum number of 100 mares. Trends have been obtained for different expression of lifetime fertility rate (LFR), obtained combining actual and predicted number of foals after the 6th reproductive season and considering different prediction methods for incomplete reproductive career (by coefficients; LFR-C; by equations; LFR-E) and arcsine transformation of the LFR-C (Arcsine LFR-C) and the LFR-E (Arcsine LFR-E).

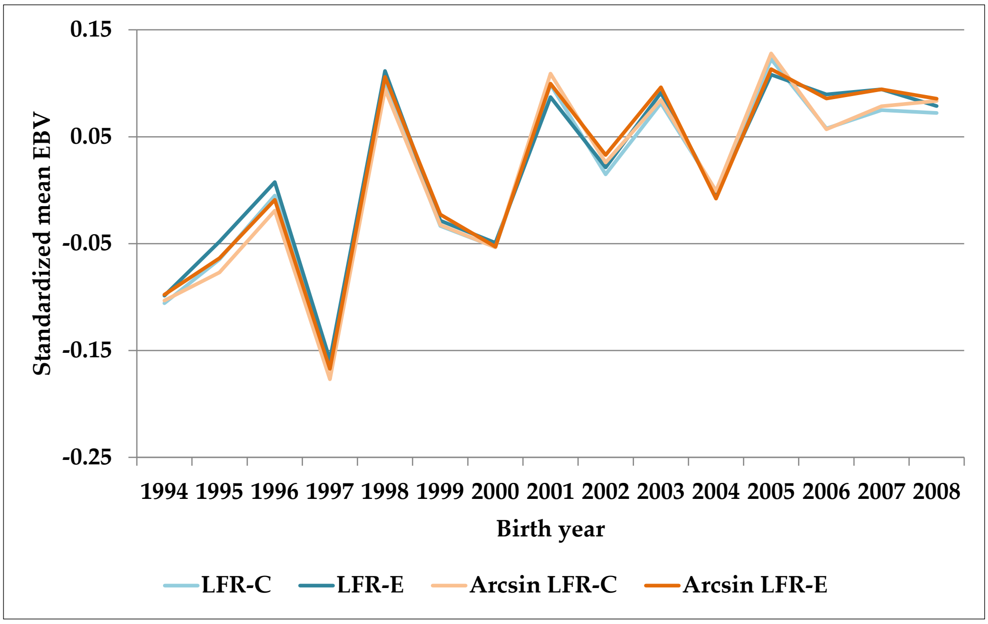

Figure 2. Genetic trends obtained from mean standardized EBVs by birth year in Italian Heavy Draught Horse stallion sires of mares with record (n = 399) tracing back 15 years from the last birth year with a minimum number of 15 stallions. Trends have been obtained for different expression of lifetime fertility rate (LFR), obtained combining actual and predicted number of foals after the 6th reproductive season and considering different prediction methods for incomplete reproductive career (by coefficients; LFR-C; by equations; LFR-E) and arcsine transformation of the LFR-C (Arcsine LFR-C) and the LFR-E (Arcsine LFR-E).
